# Detection of Highly Poisonous *Nerium oleander* Using Quantitative Real-Time PCR with Specific Primers

**DOI:** 10.3390/toxins14110776

**Published:** 2022-11-10

**Authors:** Xuanjiao Bai, Gang Wang, Ying Ren, Jianping Han

**Affiliations:** Institute of Medicinal Plant Development, Chinese Academy of Medical Sciences & Peking Union Medical College, Beijing 100193, China

**Keywords:** *Nerium oleander*, food poisoning, qPCR, specific primers, simulated forensic specimens

## Abstract

*Nerium oleander* is one of the most poisonous plants, and its accidental ingestion has frequently occurred in humans and livestock. It is vital to develop a rapid and accurate identification method for the timely rescue of oleander-poisoned patients and the investigation of poisoning cases. In this study, a specific and highly sensitive quantitative real-time PCR (qPCR)-based method was developed to identify oleander in mixture systems and simulated forensic specimens (SFS). First, a new pair of oleander-specific primers, JZT-BF/BR, was designed and validated. Then, a qPCR method was developed using the primers, and its detective sensitivity was examined. The results showed that JZT-BF/BR could specifically identify oleander in forage and food mixtures, and qPCR was capable of accurate authentication even at a low DNA concentration of 0.001 ng/μL. This method was further applied to the analysis of SFS containing different ratios of *N. oleander*. The method was confirmed to be applicable to digested samples, and the detection limit reached 0.1% (*w*/*w*) oleander in mixture systems. Thus, this study undoubtedly provides strong support for the detection of highly toxic oleander and the diagnosis of food poisoning in humans and animals.

## 1. Introduction

Poisoning cases caused by plant exposure are frequently reported by poisoning control centers worldwide [1]. The wide distribution and various applications of poisonous plants in the daily environment significantly increase the risk of food poisoning. *Nerium oleander*, also known as oleander, is a highly toxic shrub with beautiful flowers. The presence of various cardiac glycosides in all the parts of the oleander plant are the main toxins that cause acute cardiotoxicity [2,3]. Trace consumption of oleander is enough to trigger dizziness, emesis, diarrhea, arrhythmia, and even death. It was reported that just one fresh oleander leaf is able to kill a child, and five leaves can be lethal for adults [4]. Even the inhalation of oleander smoke can cause a series of poisoning symptoms [5]. Moreover, *N. oleander* is fatal to most animals. Research has verified that a cow or horse may be killed by ingesting oleander at 0.005% of the animal’s body weight [6,7]. However, because of its common distribution in the living environment as an ornamental plant, poisoning cases caused by accidental ingestion of oleander occur frequently. The Toxic Exposure Surveillance System reported that there were 785 cases of oleander poisoning in the United States in 2004 [8]. According to the investigation of 150 plant-poisoning reports in South India, oleander was suggested as the prime culprit in 65% of poisoning cases [9]. A large number of poisoning cases were also reported in the Mediterranean, where oleander is widely distributed [10,11,12]. In addition to events of human poisoning, numerous cases have also been reported in livestock due to accidental ingestion in the wild and unplanned contamination of *N. oleander* in feed [13,14,15,16]. On an Italian farm, almost 50 cows showed toxic symptoms after eating fodder accidentally mixed with dry oleander, resulting in the death of 13 cows [17]. Moreover, it has been verified that the toxic chemicals in oleander-poisoned animals can be transferred and accumulated in milk, which may pose a potential risk to consumer health and safety [17,18]. Hence, the efficient detection of *N. oleander* materials in complex mixtures is essential to protect public safety and prevent criminal incidents.

Present authentication of *N. oleander* is mainly realized by morphological identification [19], microscopic observation [20], and chemical assays [21,22]. However, in poisoning investigations, biological samples are usually complex materials, such as animal and plant residues, vomit, and stomach content. The absence of diagnostic characteristics restricts the application of morphological and microscopic identification. Chemical analyses based on thin-layer chromatography, high-performance liquid chromatography, and gas chromatography are presently the most popular methods for toxic component testing [21,23]. Nevertheless, for samples with complex and varied compositions, tedious cleanup procedures and precolumn derivatization are usually required to remove interference compounds and improve detection sensitivity, which results in a time-consuming process [24]. These challenges affect timely diagnosis in poisoning cases. Quantitative real-time PCR (qPCR), as a convenient and sensitive molecular identification method, has been widely applied in virus detection, adulterate identification, and forensic science [25,26]. For instance, because of its sensitive detection of SARS-CoV-2 in the early stages of infection, qPCR technology has been deemed the “gold standard” for clinical diagnosis [27]. In drowning research, even with only 0.0001 ng of template phytoplankton DNA, qPCR was able to provide clues for forensic investigation [25]. In addition, qPCR is commonly applied in the identification of adulterant species in highly processed products and complex mixtures [28,29]. Research has demonstrated the successful application of qPCR in the identification of questionable ingredients in herbal products and processed foods [28,30,31]. The established qPCR method was able to identify three common poisonous plants in processed food and digested samples, including *Caltha palustris*, *Ambrosia artemisiifolia*, and *Veratrum maackii var. Japonicum*, which contributed to the regulatory monitoring of commercial foods and the forensic investigation of poisoning cases [32]. However, a qPCR assay for the rapid detection of *N. oleander* has not been developed or reported to date.

Thus, in this study, we aimed to develop the first qPCR-based method capable of fine detection for the authentication of oleander-containing biomaterials in forensic examinations and diagnosis of food poisoning in humans and animals.

## 2. Results

### 2.1. Establishment of the qPCR Assay for N. oleander Detection

**Figure 1:**
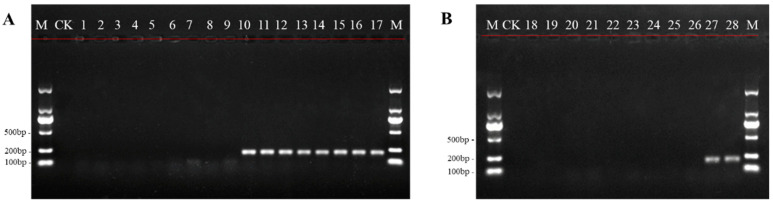
Specificity validation of JZT-BF/BR. M, DNA marker; CK, blank control using ddH_2_O as template; (**A**) 1–8, herbage samples of *Sonchus oleraceus*, *Achillea millefolium, Alcea rosea*, *Phalaris arundinacea*, *Trigonotis peduncularis, Pastinaca sativa, Medicago sativa*, and *Viola selkirkii*, respectively; 9, herbage mixture; 10, herbage mixture containing oleander; 11–17, individual oleander samples; (**B**) 18–25, vegetable samples of pak choi, lettuce, spinach, greengrocery, needle mushroom, tea tree mushroom, edible fungus, and tomato, respectively; 26, vegetable mixture; 27, vegetable mixture containing oleander; 28, oleander sample.

Based on the internal transcribed spacer (ITS) sequences of oleander and its closely related species, the primer pair JZT-BF/BR specific to *N. oleander* was obtained by sequence screening. The results of Primer-Blast indicated that the corresponding amplicon of 200 bp was only generated in *N. oleander* DNA with JZT-BF/BR (Figure S1). In order to further verify its specificity and identification ability, JZT-BF/BR was applied to distinguish oleander from herbages, vegetables, and their DNA mixtures. The results indicated that clear and bright electrophoretic bands could be observed for oleander collected from different locations, while no amplification could be visualized in the fodder and vegetable samples (Figure 1). In addition, no amplification products were observed in the mixed herbage and vegetable DNA samples. However, when oleander DNA was added to the mixed herbage and vegetable DNA, there was a single and special band of PCR products in the stained gel (Figure 1). These results fully confirmed the applicability of JZT-BF/BR and laid a good foundation for the establishment of the qPCR method.

**Figure 2:**
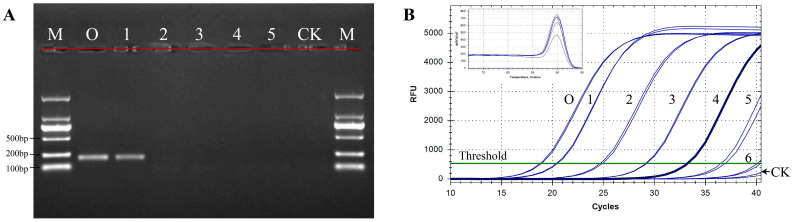
Sensitivity of conventional PCR (**A**) and the established qPCR method (**B**). M, DNA marker; CK, negative control using ddH_2_O as template; O, *N. oleander*; 1–6, *N. oleander* DNA concentrations of 10, 1, 0.1, 0.01, 0.001, and 0.0001 ng/μL, respectively.

The detection sensitivity of qPCR combined with the specific primers was explored and compared with that of conventional PCR. A total of six serial ten-fold dilutions of *N. oleander* DNA, ranging from 100 to 0.0001 ng/μL, were examined. For conventional PCR, the amplification products of DNA diluted 10-fold were successfully detected by gel electrophoresis but were not detectable when oleander DNA was diluted by 100 or more times (Figure 2A). In contrast, amplification was adequate, and obvious melting curves were observed using the qPCR method, even at concentrations of *N. oleander* DNA as low as 0.001 ng/μL after five serial ten-fold dilutions (Figure 2B). Extremely weak fluorescent signals were detected when the template was diluted six times and for the negative control at Ct > 40. According to standard qPCR cycling conditions [33], we concluded that the detection sensitivity of the qPCR method was approximately 0.001 ng/μL of oleander DNA. Additionally, the results showed that the specific melting point of *N. oleander* DNA was approximately 91.0 ± 0.5 °C. The analysis demonstrated that the assay was capable of specifically distinguishing oleander from herbage and vegetable species with high sensitivity.

### 2.2. Application of the qPCR in SFS

**Figure 3:**
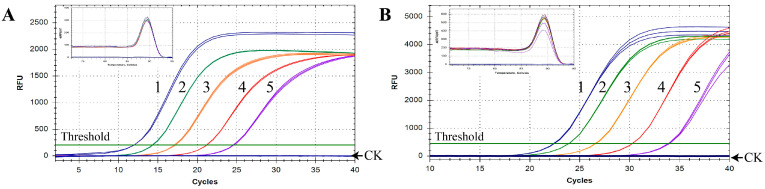
Oleander detection in SFS for animals (**A**) and humans (**B**) using qPCR with the specific primers. CK, negative control using ddH_2_O as template; 1–5, SFS containing 100%, 50%, 10%, 1%, and 0.1% *N. oleander*, respectively.

In order to estimate the identification capacity for *N. oleander* in practical applications, the established qPCR assay was implemented to detect oleander in animal and human SFS. The results showed that the amplification signal could be detected in both types of SFS, even if the content of oleander was only 0.1% (Table 1, Figure 3). Furthermore, all the animal SFS, including those containing trace amounts of oleander, were detected using the qPCR method with Cq values under 30, which indicated that the amplification efficiency of *N. oleander* DNA was pretty high (Figure 3A). It was found that the Cq values of human SFS were higher than those of animal SFS with the same content of oleander, meaning that the additional boiling treatment used in the preparation of human SFS affected the detectability of *N. oleander* DNA (Figure 3B). In spite of this, obvious amplification curves with good reproducibility were observed for human SFS containing 0.1% oleander, which confirmed the reliability of the qPCR method in the identification of trace amounts of oleander among complex mixtures. In addition, in all samples, the obvious peak of the melting curves at a specific temperature further indicated the existence of oleander. Moreover, the occurrence of only one melting curve peak illustrated the specificity of the qPCR method without the presence of non-specific amplicons or primer-dimer formation (Figure 3). In summary, the results validated the excellent performance of the newly developed qPCR method in detecting oleander in SFS.

## 3. Discussion

### 3.1. Significance of the Development of qPCR for N. oleander Detection

*N. oleander* is commonly cultivated at the roadside and in parks and private gardens because of its beautiful flowers and excellent air-purifying capabilities [2,18,20,34]. Disturbingly, poisoning cases caused by the mistaken intake of oleander or oleander-containing materials increasingly occur. The economic losses and safety issues due to human and livestock poisoning cannot be ignored [11,12]. In this study, we developed a qPCR method to more effectively identify *N. oleander* and oleander-containing mixtures for the purpose of *N. oleander* poisoning investigations. A pair of ideal primers at the species level is fundamental to guarantee the specificity of a qPCR method. The bioinformatics analysis of the designed primers, JZT-BF/BR, indicated that they could only be bound to the ITS region of oleander DNA, which provided theoretical support for the specificity of the qPCR method we constructed (Figure S1). In addition, a total of 16 samples, including familiar herbages and vegetables, were collected to investigate the specificity of JZT-BF/BR. The results showed that there was no visible amplification for the eight edible animal fodders, eight vegetables in the human diet, and mixtures made of them. In contrast, bright electrophoretic bands appeared in the seven batches of oleander individually collected from three different provinces, as well as the oleander-positive mixtures prepared with fodder or vegetables. The detection capacity of JZT-BF/BR in complex mixtures was further confirmed to be dependable. Due to the extremely high toxicity of oleander, even trace amounts via oral administration are sufficient to cause poisoning. Therefore, the sensitivity of the detection method is critical for diagnostic accuracy. Indeed, previous studies have shown that qPCR is suitable for the analysis of very small amounts of DNA and far more sensitive than conventional PCR [35]. The sensitivity experiments in our study indicated that even 0.001 ng/μL of oleander DNA was sufficient to be detected by qPCR, which was significantly more sensitive than conventional PCR (Figure 2). Similar sensitivity was reported for a qPCR assay that detected 0.001 ng of pork DNA using serial dilutions of pork genomic DNA extracted from cooked pork [29]. Furthermore, the results of poisoning investigations can be obtained from intuitive fluorescence signals using qPCR, which is more rapid and convenient since it does not require agarose gel detection nor sequencing analyses [36]. Moreover, as a DNA-based identification method, qPCR can overcome the deficiency of chemical methods in identifying different species with similar chemical profiles to confirm the sources of toxins. For instance, through the detection of stomach contents from the dead using qPCR, Sakurada et al. [37] accurately narrowed down the lethal matter to the colchicine-containing plant *Gloriosa superba*. Thus, the qPCR method developed in the current study could serve as a highly sensitive assay to precisely identify oleander and oleander-containing materials, thereby providing a powerful approach to determine the poisoning cause in cardiac glycoside-triggered emergencies.

### 3.2. Detection of Oleander-Containing Materials with qPCR

Previous studies have verified that oleander can be quickly absorbed in the gastrointestinal tract and results in immediately toxic effects after oral administration [3]. The stomach contents from patients or the dead could be suitable for determining oleander poisoning [22]. However, to date, clinical analyses in oleander toxicity cases have mainly focused on the detection of oleandrin in blood, serum, liver, and heart tissues [17,38]. Because of the complex and variable composition of gastric contents, it is hard to identify oleander in digested samples by chemical methods or morphological observation. Thus, establishing more effective methods for the authentication of *N. oleander* in complex mixture systems is urgently needed. In this study, a sensitive and convenient qPCR method was developed for the detection of *N. oleander* and oleander-containing materials, which was proven to be specific to *N. oleander*. In order to assess the practical identification of *N. oleander* in poisoning diagnosis, we applied the constructed qPCR assay to ten batches of SFS. The results showed that the qPCR method successfully detected 0.1% oleander in SFS after boiling treatment and lengthy digestion. The same detection limit was reported for a qPCR method that was able to detect 0.1% target species in wheat flour and was effective for forensic investigations of patients who had ingested poisonous plants [32]. In addition, the typical unimodal peak of the melting curve illustrated unambiguous species distinction in complex samples with multiple ingredients. Interestingly, at the same content of oleander, the Cq values of human SFS after cooking and digestion treatments were obviously higher than those of animal SFS, which were only digested. This difference is likely because the additional boiling treatment reduced the amplification efficiency of the target DNA. In addition to boiling, processes such as high temperature, grinding, or pH change can vastly decrease the integrity of DNA. Moreover, the length of digestion time was also found to affect the detection efficiency of qPCR methods [32]. Previous studies verified that with prolonged processing time, the amplification efficiency of DNA fragments longer than 200 bp was gradually reduced [39,40]. Compared to identification using full barcode regions, the application of qPCR presents obvious advantages for the analysis of degraded samples due to the short amplicon size [41]. In our study, the amplified length of 200 bp greatly overcomes the challenges posed by the forensic examination of materials with serious DNA degradation. In general, qPCR technology can successfully detect trace quantities of poisonous plants in SFS and has potential uses in the diagnosis of food poisoning.

## 4. Conclusions

In this study, a qPCR method was first proposed to detect oleander-containing materials for forensic investigation and poisoning diagnosis in humans and animals. The method was verified to be specific and sensitive for oleander in oleander-containing mixtures and was successfully applied to identify trace amounts of oleander in SFS. The established method could help achieve a precise diagnosis of oleander poisoning and overcome the difficulty of detecting the target species in complex mixtures with unknown and multiple ingredients. Furthermore, the method can serve as a complementary method to chemical analysis to help trace the source of toxins, especially in forensic science. Further development of this assay for other poisonous plants could greatly expand the application of toxicant DNA authentication. Undoubtedly, the utility of qPCR in poison identification will help further prevent toxic exposure, determine the poisoning cause, and significantly protect the safety of animals and humans.

## 5. Materials and Methods

### 5.1. Collection and Preparation of Materials

Material Collection: Seven individual *N. oleander* samples were collected from Jiangsu, Hainan, and Beijing in China (Table 2). To prepare the simulated forensic specimens (SFS), common herbages and vegetables were collected. The herbage materials for *Sonchus oleraceus*, *Achillea millefolium, Alcea rosea*, *Phalaris arundinacea*, *Trigonotis peduncularis, Pastinaca sativa, Medicago sativa*, and *Viola selkirkii* were gathered to prepare animal SFS samples. Similarly, vegetables, including pak choi, lettuce, spinach, greengrocery, needle mushroom, tea tree mushroom, edible fungus, and tomato, were purchased from the market to imitate SFS of poisoned humans. Additionally, a total of 71 ITS sequences of *N. oleander* and other Apocynaceae species closely related to oleander were downloaded from GenBank (https://www.ncbi.nlm.nih.gov/) accessed on 15 August 2022, which were used as references for the development of the oleander-specific primers. The accession numbers of downloaded sequences are shown in Table S1.

Simulated forensic specimen preparation: To simulate the gastric contents of poisoned individuals, animal and human SFS were prepared using herbages and vegetables, respectively. For the preparation of animal SFS, first, an equal amount of each herbage sample was mixed together to obtain the herbage mixture (HM). Then, HM was spiked with 100%, 50%, 10%, 1%, and 0.1% (*w*/*w*) oleander. The resulting mixtures were incubated in 50 mL simulated gastric fluid (Coolaber Co., Beijing, China) at 37 °C for 4 h to obtain the SFS of animals. Similarly, to prepare the SFS of humans, the food mixture (FM) was prepared using a balanced mixture of vegetables, which was spiked with 100%, 50%, 10%, 1%, and 0.1% (*w*/*w*) oleander. Additionally, in order to simulate the cooking process, the obtained mixtures were first boiled at 100 °C for 30 min and then incubated in simulated gastric fluid (Coolaber Co., Beijing, China) at 37 °C for 4 h.

### 5.2. DNA Extraction

Plant samples: Approximately 20–30 mg of each sample was collected in a centrifuge tube and ground into fine powder using a ball-milling machine (Restch Co., Haan, Germany) at a frequency of 30 Hz for 2 min. Then, DNA extraction was performed using the Plant Universal Genomic DNA kit (Tiangen Biotech Beijing Co., Beijing, China), according to manufacturer’s instructions.

SFS samples: Each SFS sample was centrifuged at 7500 rpm for 5 min, and the supernatant was discarded. About 30–40 mg of sediment was collected in a centrifuge tube and milled into a paste using a ball-milling machine (Restch Co., Haan, Germany). In each tube, 700 μL of pre-wash buffer (100 mM Tris-HCl, pH 8.0; 20 mM EDTA, pH 8.0; 700 mM NaCl; 2% PVP-40; 0.4% β-mercaptoethanol) was added to wash the precipitate, and the tube was centrifuged at 7500 rpm for 5 min at room temperature to remove the supernatant. This step was repeated several times until the supernatant was clear and colorless. Then, the genomic DNA was extracted from the remaining precipitate using the Plant Universal Genomic DNA Kit (Tiangen Biotech Beijing Co., Beijing, China), according to manufacturer’s instructions.

### 5.3. Design and Verification of N. oleander-Specific Primers

We initially tried to obtain the ITS sequences from the seven individual oleander samples using the universal primers ITS-5F/4R and ITS-2F/3R, respectively [42]. However, it was found that the sequencing results were messy, and accurate barcode sequences were unavailable. Thus, to collect reliable sequence information, a new primer pair, ITS-F (TGCGGAAGGATCATTGTCGA)/R (TGCGTTCAAAAACTCGATGG), was designed based on the downloaded ITS sequences of oleander using CodonCode Aligner 3.7.1 (CodonCode Co., Centerville, MA, USA). Then, amplification and sequencing were performed using the designed primers, referring to the universal procedure to obtain the final ITS sequences. The ITS sequences obtained from experiments and GenBank for oleander and its closely related species were aligned using MEGA-X software [43] to search for oleander-specific loci, and the candidate primers were screened out using Primer Premier 6.0 (Premier Co., Palo Alto, CA, USA) in these regions. The specificity of the primers was tested using the Primer-Blast tool of the National Center for Biotechnology Information (NCBI, Bethesda, MD, USA. https://blast.ncbi.nlm.nih.gov/Blast.cgi) assessed on 27 August 2022, and JZT-BF (5′-CTCGTTTATCCTCGGGCGT-3′)/JZT-BR (5′-AGATTCGACTGGCGCCTTT-3′) were finally selected as oleander-specific primers. To further verify their specificity and applicability, the primers were used to amplify the individual oleander samples, forages, and vegetables. The reaction was carried out in a 25 µL system containing 1.0 μL of JZT-BF/BR primers (2.5 µM), 12.5 μL of 2xTaq PCR Master Mix (HT-biotech Co., Beijing, China), 2.0 μL of template DNA, and 8.5 μL of double-distilled water. The reaction conditions were set as follows: initial denaturation at 97 °C for 5 min; followed by 30 cycles of denaturation at 97 °C for 30 s, annealing at 55 °C for 45 s, and extension at 72 °C for 30 s; and a final elongation step at 72 °C for 7 min. The PCR products were examined by 1% (*w*/*v*) agarose gel electrophoresis and visualized using a gel imaging system (Bio-Rad Co., Hercules, CA, USA).

### 5.4. Sensitivity Test of qPCR

To explore the detection sensitivity of qPCR combined with the specific primers, the *N. oleander* DNA extracted from fresh leaves was ten-fold serially diluted 1 to 6 times to achieve different ratios. The concentration of diluted DNA was measured using a NanoDrop 2000C spectrophotometer (Thermo Fisher Scientific, Waltham, MA, USA). All samples were analyzed using both conventional PCR and qPCR methods. The reaction conditions for the conventional PCR method and analysis of the results were performed as described in Section 5.3. The qPCR assay was carried out in a 25 μL system containing 12.5 μL of SYBR Premix Ex Taq™ (Takara Bio, Tokyo, Japan), 1.0 μL of JZT-BF/BR primers (2.5 µM), 2.0 μL of template DNA, and 8.5 μL of double-distilled water. Three replicates were analyzed for each sample. The reactions were carried out with a Real-Time PCR instrument (Bio-Rad Co., Hercules, CA, USA) as follows: 95 °C for 30 s, followed by 40 cycles of 95 °C for 30 s, 56 °C for 45 s, and 72 °C for 30 s. Subsequently, melting curve analysis was performed by raising the reaction temperature from 65 °C to 95 °C at a rate of 0.5 °C/s. Data analysis for qPCR was conducted using CFX Manager^TM^ 3.1 Software (Bio-Rad Co., Hercules, CA, USA).

### 5.5. Oleander Detection in Simulated Forensic Samples

The established qPCR assay was used to detect *N. oleander* in SFS to estimate the applicability of the method in oleander poisoning diagnosis. Reaction conditions were in accordance with those described in Section 5.4. All experiments were repeated three times to validate the repeatability of the method.

## Figures and Tables

**Table 1:** The detection results of SFS samples using qPCR in this study.

Type	Oleander Content (*w*/*w*)	Boiling	Digestion	Detection
Animals	100%	-	37 °C, 4 h	√
	50%	-	37 °C, 4 h	√
	10%	-	37 °C, 4 h	√
	1%	-	37 °C, 4 h	√
	0.1%	-	37 °C, 4 h	√
Humans	100%	100 °C, 30 min	37 °C, 4 h	√
	50%	100 °C, 30 min	37 °C, 4 h	√
	10%	100 °C, 30 min	37 °C, 4 h	√
	1%	100 °C, 30 min	37 °C, 4 h	√
	0.1%	100 °C, 30 min	37 °C, 4 h	√

**Table 2:** The information of individual oleander samples in this study.

Sample No.	Voucher No.	Resource	GenBank Accession	Sequence Type
O1	JZT2101	Suqian, Jiangsu	OP658836	ITS
O2	JZT2102	Suqian, Jiangsu	OP658837	ITS
O3	JZT2103	Suqian, Jiangsu	OP658838	ITS
O4	JZT2104	Muyang, Jiangsu	OP658839	ITS
O5	JZT2105	Suqian, Jiangsu	OP658840	ITS
O6	JZT2106	Haidian, Beijing	OP658841	ITS
O7	JZT2107	Haikou, Hainan	OP658842	ITS

## Data Availability

The data presented in this study are openly available in https://www.ncbi.nlm.nih.gov/ with the GenBank accession numbers in Table 2.

## Supplementary Materials

The following supporting information can be downloaded at: https://www.mdpi.com/article/10.3390/toxins14110776/s1, Figure S1: Primer-BLAST result of the sequences of JZT-BF/BR; Table S1: Information of the 71 ITS sequences of *N. oleander* and other Apocynaceae species closely related to oleander downloaded from GenBank.
