# Supplementary material for: Detection of Highly Poisonous Nerium oleander Using Quantitative Real-Time PCR with Specific Primers
Source: Toxins (Basel). 2022 Nov 10;14(11):776. doi: 10.3390/toxins14110776 (PMC9696062; doi:10.3390/toxins14110776)
Supplement: Supplementary file 1 [file toxins-14-00776-s001.zip › toxins-2012150-supplementary.pdf]

toxins

MDPI

Article

Detection of Highly Poisonous *Nerium oleander* Using Quantitative Real-Time PCR with Specific Primers

Xuanjiao Bai, Gang Wang, Ying Ren, and Jianping Han

Table S1. Information of the 71 ITS sequences of *N. oleander* and other Apocynaceae species closely related to oleander downloaded from GenBank.

No.	Species	GenBank accession
1	*Aganosma cymosa*	MF350550、51、52
2	*Aganosma schlechteriana*	MF350555
3	*Aganosma wallichii*	MF350553-4
4	*Alstonia scholaris*	MG757606
5	*Amsonia elliptica*	MK039532
6	*Amsonia tomentosa*	MF963953
7	*Anodendron affine*	KP092767
8	*Apocynum hendersonii*	KP152520、21、22、23、24、25
9	*Apocynum pictum*	KP152516、17、18
10	*Apocynum venetum*	MN722099
11	*Asclepias syriaca*	JF312046
12	*Aspidosperma excelsum*	MK039533
13	*Atherolepis wallichii*	KT318133
14	*Carissa macrocarpa*	MK039534
15	*Condylocarpon isthmicum*	MK039535
16	*Cycladenia humilis var. humilis*	MF964023
17	*Dyera costulata*	MK039536
18	*Epigynum auritum*	MF350548-49
19	*Epigynum cochinchinense*	MF350528-29
20	*Epigynum griffithianum*	MF350536
21	*Epigynum ridleyi*	MF350535
22	*Gongronema taylorii*	MW226529
23	*Gonioma kamassi*	MK039537
24	*Gonolobus suberosus*	KY707178
25	*Hancornia speciosa*	MK039538
26	*Holarrhena pubescens*	MN257723, MT106638
27	*Marsdenia truncata*	MW226533
28	*Matelea parvifolia*	MF964167
29	*Melodinus cochinchinensis*	MK039539
30	*Nerium oleander*	KP764848, MH548390-91, MT106635
31	*Parsonsia crebriflora*	KY483911
32	*Parsonsia eucalyptophylla*	KY483912
33	*Parsonsia ferruginea*	KY483913
34	*Parsonsia lenticellata*	KY483914
35	*Parsonsia longiflora*	KY483915
36	*Parsonsia oligantha*	KY483916
37	*Periploca sepium*	MN721986

38	*Plumeria alba*	HQ130658
39	*Plumeria rubra*	HQ130659
40	*Strophanthus divaricatus*	MH844621, MH844589, MH558640
41	*Trachelospermum asiaticum*	MF350546, MH808525, MF350541
42	*Trachelospermum axillare*	MF350542-3
43	*Trachelospermum jasminoides*	JF708198, MH711772
44	*Vallaris solanacea*	HQ386700
45	*Vinca major*	MK039541
46	*Vincetoxicum fuscatum*	MT276344
47	*Vincetoxicum hirundinaria subsp. adriaticum*	MT276362
48	*Vincetoxicum pannonicum*	MT276345
49	*Vincetoxicum stauntonii*	EU591970

Input PCR template none
Specificity of primers Target templates were found in selected database: Nucleotide collection (nt) (Organism limited to Viridiplantae)
Other reports ▷Search Summary

— Detailed primer reports

Primer pair 1

	Sequence (5'->3')	Length	Tm	GC%	Self complementarity	Self 3' complementarity
Forward primer	CTCGTTTATCCTCGGGCGT	19	59.56	57.89	2.00	0.00
Reverse primer	AGATTCGACTGGCGCCTTT	19	59.70	52.63	6.00	4.00

Products on target templates

>MH548390.1 Nerium oleander isolate 1766 internal transcribed spacer 1, partial sequence; 5.8S ribosomal RNA gene, complete sequence; and internal transcribed spacer 2, partial sequence

```
product length = 198
Forward primer  1    CTCGTTTATCCTCGGGCGT  19
Template        85   ...................  103

Reverse primer  1    AGATTCGACTGGCGCCTTT  19
Template        282  ...................  264
```

>KP764848.1 Nerium oleander voucher BOL:Bruyns s.n. promoter region; 18S ribosomal RNA gene, partial sequence; internal transcribed spacer 1 and 5.8S ribosomal RNA gene, complete sequence; and internal transcribed spacer 2, partial sequence

```
product length = 200
Forward primer  1    CTCGTTTATCCTCGGGCGT  19
Template        56   ...................  74

Reverse primer  1    AGATTCGACTGGCGCCTTT  19
Template        255  ...................  237
```

>HE602380.1 Nerium oleander genomic DNA containing ITS1, 5.8S rRNA gene, ITS2, specimen voucher Mateos, MA. 7113

```
product length = 199
Forward primer  1    CTCGTTTATCCTCGGGCGT  19
Template        621  ...........A.......  603

Reverse primer  1    AGATTCGACTGGCGCCTTT  19
Template        423  ...................  441
```

>MZ675655.1 Nerium oleander voucher JBRspecimen-200004 internal transcribed spacer 1, partial sequence; 5.8S ribosomal RNA gene, complete sequence; and internal transcribed spacer 2, partial sequence

```
product length = 199
Forward primer  1  CTCGTTTATCCTCGGGCGT  19
Template        1  .........A.A.......  19

Reverse primer  1    AGATTCGACTGGCGCCTTT  19
Template        199  ...................  181
```

Figure S1. Primer-BLAST result of the sequences of JZT-BF/BR.
